# Editorial: Novel therapeutics for urological cancers

**DOI:** 10.3389/fphar.2025.1771902

**Published:** 2026-01-07

**Authors:** Shuai Tang, Benyi Li

**Affiliations:** 1 Department of Urology, University of Kansas Medical Center, Kansas City, KS, United States; 2 Department of Urology, The Third Central Hospital of Tianjin, Tianjin, China; 3 College of Medicine, Nankai University, Tianjin, China

**Keywords:** combination therapy, ferroptosis, gut microbiota metabolites, minimally invasive ablation, prostate cancer, urological cancers

Urologic oncology is undergoing a structural shift, and systemic therapies continue to advance, yet tumor heterogeneity and acquired resistance still constrain durable benefit. In parallel, local and minimally invasive approaches are expanding their reach, bringing organ preservation and functional protection into routine decision-making. In this Research Topic, “novel therapeutics” is used in a broad, practical sense, work that links biological rationale to interventions that can be evaluated in patients and implemented in routine care. The contributions span metabolite-linked mechanisms, ferroptosis-oriented therapeutics, regimen optimization in advanced prostate cancer, refinements in renal tumor ablation, and organ-sparing strategies in selected, evidence-scarce settings.

The microbiome-metabolite axis is clinically relevant because it connects interpretable biology with potential intervention windows (Yang et al., 2023). Two studies in this Topic provide complementary, directionally opposed, but jointly informative, evidence that argues against simplistic “good versus bad metabolite” labeling. One study places trimethylamine N-oxide (TMAO) into a defined signaling framework, suggesting that a p38/HMOX1 axis promotes a more invasive phenotype and aligns these signals with *in vivo* observations (Zhou et al.). In contrast, another study reports that phenylacetylglutamine (PAGln) suppresses prostate cancer progression by upregulating CCNG2 and dampening Wnt/β-catenin signaling (Lv et al.). Considered together, these works shift the practical question toward stratification: which patient subsets demonstrate stable, reproducible metabolite–pathway signatures, and which signatures can realistically be translated into clinically usable risk or response markers.

Ferroptosis offers a pharmacologic entry point distinct from canonical apoptosis by reframing vulnerability around iron-dependent lipid peroxidation (Dixon et al., 2012). The study of cepharanthine hydrochloride advances this concept in prostate cancer by linking ferroptosis-associated phenotypes to specific defense and regulatory components, including GPX4/FSP1 and lipid-peroxidation-related regulators such as ACSL4 and DHODH (Guan et al.). As the field moves from mechanism to medicine, translation will hinge on actionable readouts, pharmacodynamic markers, a defined safety window, and a rational combination strategy, so ferroptosis induction can be tested as a targeted approach rather than applied indiscriminately (Wahida and Conrad, 2025). Importantly, ferroptosis-oriented strategies will likely be evaluated not as stand-alone “replacement therapies,” but as sensitizers or complements within existing treatment backbones, where incremental benefit must be weighed against additive toxicity and biological plausibility.

In metastatic castration-resistant prostate cancer (mCRPC), patients frequently enter chemotherapy after sequential androgen receptor axis-targeted therapies; at that stage, the clinical question is whether intensification still adds meaningful incremental benefit in a “pre-selected” and often more resistant population. The comparative study of enzalutamide plus docetaxel versus docetaxel alone speaks directly to this real-world decision point, reporting signals consistent with improved disease control and symptom relief with manageable tolerability after sequential ARAT failure (Zhang et al.). The key contribution is not to elevate combination therapy to default status, but to sharpen decision-making: identifying who is most likely to benefit, how to manage cumulative toxicity, and where to place the combination within a broader sequencing framework consistent with contemporary guideline-based care (Fizazi et al., 2023). Moving forward, clearer definitions of prior exposure, clinically meaningful endpoints (including symptom control), and prospective validation will be essential for determining whether such combinations can be generalized beyond specific practice settings.

For renal cell carcinoma, the central challenge is achieving complete ablation without unacceptable collateral injury, particularly for larger or high-risk lesions. The review on adjunctive techniques emphasizes procedural adjuncts, pre-ablation embolization, hydro-dissection, and other protective maneuvers that can improve lesion conspicuity, energy delivery, and adjacent-structure protection (Torres et al.). To translate technical ingenuity into broadly adoptable practice, the next step is standardization: consistent technique descriptors, harmonized outcome reporting, and higher-quality comparative evidence to define where benefit is largest and most reproducible (Clark et al., 2006; Powles et al., 2024).

The case report addressing a massive, muscle-invasive inflammatory myofibroblastic tumor (IMT) of the bladder proposes a staged bladder-sparing strategy, *en bloc* transurethral resection followed by laparoscopic partial cystectomy, supported by pathology and follow-up (Chu et al.). Its transferable value lies in clarifying decision points: defining candidacy, margin assessment, surveillance intensity, and how to articulate the trade-off between oncologic control and functional preservation in a disease space where management remains heterogeneous (Hage et al., 2024). Although benign prostatic hyperplasia is not a malignancy, the nobiletin study provides a methodologically relevant example of mechanism-driven pharmacology in urologic disease: integrating *in vitro* and *in vivo* evidence to connect cell-cycle regulation, signaling modulation, and androgen-axis involvement to measurable antiproliferative effects (Song et al.). Within this Topic, it serves as a reminder that multi-target regulation and natural-product pipelines can produce hypotheses and tool compounds that may inform oncology-adjacent translational work, provided they are advanced with rigorous readouts and well-defined clinical questions.

Taken together, these papers highlight three recurring themes: translating mechanism-based signals into clinically reproducible stratification, refining combination or sequencing strategies in advanced disease with clearer eligibility and toxicity frameworks, and improving the reliability of minimally invasive local therapy through standardization of technical descriptors and outcome reporting. These priorities will largely determine whether promising biological or technical advances can be carried forward into more comparable evidence and broader clinical adoption.
